# Protective role of oleic acid against cardiovascular insulin resistance and in the early and late cellular atherosclerotic process

**DOI:** 10.1186/s12933-015-0237-9

**Published:** 2015-06-10

**Authors:** Liliana Perdomo, Nuria Beneit, Yolanda F. Otero, Óscar Escribano, Sabela Díaz-Castroverde, Almudena Gómez-Hernández, Manuel Benito

**Affiliations:** Biochemistry and Molecular Biology Department, School of Pharmacy, Complutense University of Madrid, Madrid, Spain; Health Research Institute of San Carlos Clinic Hospital (IdISSC), Madrid, Spain; CIBER of Diabetes and Associated Metabolic Diseases, Madrid, Spain

**Keywords:** Fatty acids, Atherosclerosis, Insulin resistance

## Abstract

**Background:**

Several translational studies have identified the differential role between saturated and unsaturated fatty acids at cardiovascular level. However, the molecular mechanisms that support the protective role of oleate in cardiovascular cells are poorly known. For these reasons, we studied the protective role of oleate in the insulin resistance and in the atherosclerotic process at cellular level such as in cardiomyocytes (CMs), vascular smooth muscle cells (VSMCs) and endothelial cells (ECs).

**Methods:**

The effect of oleate in the cardiovascular insulin resistance, vascular dysfunction, inflammation, proliferation and apoptosis of VSMCs were analyzed by Western blot, qRT-PCR, BrdU incorporation and cell cycle analysis.

**Results:**

Palmitate induced insulin resistance. However, oleate not only did not induce cardiovascular insulin resistance but also had a protective effect against insulin resistance induced by palmitate or TNFα. One mechanism involved might be the prevention by oleate of JNK-1/2 or NF-κB activation in response to TNF-α or palmitate. Oleate reduced MCP-1 and ICAM-1 and increased eNOS expression induced by proinflammatory cytokines in ECs. Furthermore, oleate impaired the proliferation induced by TNF-α, angiotensin II or palmitate and the apoptosis induced by TNF-α or thapsigargin in VSMCs.

**Conclusions:**

Our data suggest a differential role between oleate and palmitate and support the concept of the cardioprotector role of oleate as the main lipid component of virgin olive oil. Thus, oleate protects against cardiovascular insulin resistance, improves endothelial dysfunction in response to proinflammatory signals and finally, reduces proliferation and apoptosis in VSMCs that may contribute to an ameliorated atherosclerotic process and plaque stability.

**Electronic supplementary material:**

The online version of this article (doi:10.1186/s12933-015-0237-9) contains supplementary material, which is available to authorized users.

## Background

Elevated circulating levels of saturated free fatty acids (SFAs; *e.g.* palmitate) induce inflammatory responses and cause insulin resistance in peripheral tissues. By contrast, mono- or poly-unsaturated FFAs (MUFAs or PUFAs) protects against SFAs. Elevated saturated FFAs can induce inflammation and insulin resistance, through several mechanisms including diacylglycerol-mediated activation of protein kinase Cθ [1] and activation of Toll-like receptors (TLR) [2]. Both mechanisms lead to the activation of the proinflammatory transcription factor nuclear factor-kappa B (NF-κB), which has been linked to fatty acid-induced impairment of insulin action in skeletal muscle [3, 4]. Once activated, NF-κB regulates the expression of multiple inflammatory mediators, including IL-6, TNF-α and other factors implicated in the metabolic alterations. IL-6 strongly correlates with insulin resistance and type 2 diabetes and its plasma levels are increased in patients with obesity and type 2 diabetes [5]. Moreover, NF-κB also regulates the expression of genes involved in the early and late atherosclerotic process and its instability, such as endothelial nitric oxide synthase (eNOS), adhesion molecules (*e.g.* ICAM-1), monocyte chemotactic protein 1 (MCP-1), and plasminogen activator inhibitor-1 (PAI-1) [6–8]. On the other hand, activated NF-κB also might be implicated in JNK-1/2 activation and induces insulin resistance in several tissues [9].

Various translational studies have identified the differential role of saturated and unsaturated fatty acids and their effects at cardiovascular level [10, 11]. MUFAs, as oleic acid, improve lipid profile [12], maintain a balance of body weight [13] and prevent palmitate-induced mitochondrial dysfunction, insulin resistance and inflammatory signalling in neuronal cells [14] and skeletal muscle [15]. However, the underlying molecular mechanisms of the protective role of oleate in cardiovascular cells are poorly known. Thus, we have studied the protective role of oleate in insulin resistance and in the early and late atherosclerotic process and its instability at the cellular level. To assess this objective, we analyzed the insulin signalling in cardiovascular cells such as endothelial cells (ECs), vascular smooth muscle cells (VSMCs) and cardiomyocyte cells (CMs), the latter two cell lines were generated by our laboratory. Secondly, we have explored the differential molecular mechanisms of oleate and palmitate on JNK-1/2 or NF-κB signalling pathways. Furthermore, we analysed the protective role of oleate in the expression of inflammatory and proatherogenic markers as well as in the proliferation and apoptosis of VSMCs. Our data provide a new insight on the differential effect of oleate versus palmitate giving support to recent findings that highlight the beneficial effects of oleic acid as an essential component of the Mediterranean diet at cardiovascular level.

## Methods

### Cell lines

Primary VSMCs were obtained from thoracic aorta arteries of three male of 8 week-old wild-type mice. Anesthetized mice (Avertin, 250 mg/kg, ip) were saline perfused and thoracic aorta arteries were submitted to collagenase dispersion and primary culture as previously described [16]. Other cell line also generated in our laboratory was neonatal cardiomyocytes from hearts of eight neonatal mice (WT CMs). Hearts were dispersed with collagenase type II (1.2 mg/ml, Whorthington) and treated with DNAsa I, grade II (10 μg/ml, Roche) at 37 °C for 20 min. Cells were grown with high glucose DMEM (Life Technologies), Fetal Bovine Serum (5 %), Horse Serum (10 %), HEPES (20 mM), cardiotrophin I (R&D Systems 0.2 ng/ml), insulin (1 μM) and a mixture of antibiotics and antifungals. Cells were seeded in plates pretreated with collagen type I (Collagen from rat tail, Roche Applied Sciences) to facilitate adhesion of cardiomyocytes. So, both primary cultures of WT VSMCs or WT CMs were infected by retroviral infection (viral particles containing pBabe retroviral vector encoding of SV40 Large T antigen) and selected with 1 μg/mL puromycin for 3 weeks.

Mouse endothelial cells (ECs) were purchased from ATCC (CRL-2167). These cells were cultured with high-glucose DMEM (ATCC), with L-glutamine (4 mM), NaHCO_3_ (1.5 g/L) and was supplemented with inactivated Horse Serum (10 %).

Cell lines were cultured with 10 % FBS-DMEM and were serum-starved for 4–5 h for insulin signaling and proliferation studies or 18 h for studies of mRNA expression, and then incubated with the corresponding stimuli. For *in vitro* experiments, we have used insulin (1, 10 and 100 nmol/L), TNF-α (10 ng/mL, Sigma), angiotensin II (1 μmol/L, Sigma), oleic acid (0.6 -1 mmol/L, Sigma) and palmitic acid (0.4 - 1 μmol/L, Sigma). Moreover, we used an NF-κB inhibitor as parthenolide (10^−5^ M for 90 min).

Anna-Maria Ordelheide (Helmholtz Center Munich, Germany) kindly provided the protocol for the preparation and dissolution of fatty acids used in this study. First, a standard solution of palmitic acid (PA, 200 mM (Sigma)) was dissolved in absolute ethanol and a standard oleic acid (OA, 100 mM (Sigma)) dissolved in NaOH solution (0.1 M) was performed. Additionally, a buffer containing 20 % bovine serum albumin (BSA) / Krebs-Ringer-HEPES pH 7.4, with which the intermediate fatty acid solution is prepared to 5 mM. The solution of conjugated fatty acids is prepared minutes before experimentation left stirring at 37 °C until complete dissolution of the fatty acids are observed to prevent oxidation of these. Finally, the necessary amount of the conjugate acid (5 mM)-20 % BSA, the average fatty acid conjugation which involves low glucose DMEM, BSA (2.5 %) and FBS (0.5 %) is added. This protocol maintain the proportions 5:1 BSA-fatty acid which allows adequate transport and bioavailability of the fatty acid to the cell. In all control cells, we used BSA (2.5 %) and FBS (0.5 %) to be able to compare with OA/PA stimulations.

### Western blot

Western blot analyses were performed on protein extracts from CMs, ECs and VSMCs as previously described [17]. The antibodies used were, SV40 TAg and IκBα from Santa Cruz Biotechnology, p53 and TnT from Calbiochem, phospho-AKT (T308), AKT, p-p70S6K (Ser389), p70S6K, p-AMPK (T172), AMPK, p-JNK-1/2 (T183/Y185), Cleaved Caspase 3, p-p42/44 (S202/T204) and p42/44 from Cell Signalling, p-IRS-1 from Millipore and p-Tyr, PAI-1, α- and β-actin and α-tubulin from Sigma Corp.

### Immunoprecipitation

To obtain total cell lysates, cells from supernatants were collected by centrifugation at 2000 × g for 5 min at 4 °C. Attached cells were scrapped off in ice-cold PBS, pelleted by centrifugation at 4000 × g for 10 min at 4 °C, and resuspended in lysis buffer. Samples of cell lysates of VSMCs were sonicated 30 s at 1.5 mA, and lysates were clarified by centrifugation at 12,000 × g for 10 min. For immunoprecipitation, 150–200 μg of protein were immunoprecipitated at 4 °C with the IRS-1 antibody and isotype control serum. The immune complexes were collected on protein A-agarose and submitted to SDS-PAGE to check its phosphorylation in Tyr and Ser residues.

### Proliferation assays

Briefly, 10^4^ cells in 1 mL of complete medium were seeded into each well of an uncoated 96-well plate. The following day, the cells were serum deprived for 5 h and stimulated with TNF-α, angiotensin II or palmitate for 24 h with or without pretreatment with oleic acid for 2 h. After that, the rate of cellular proliferation was evaluated using a cell proliferation ELISA BrdU kit (Roche Applied Science). The incubation with BrdU labeling solution was for 18 h.

### Cell cycle

After treating cells in culture with the corresponding stimulus, the supernatant cells were collected and the attached cells were lifted with trypsin. The cells were centrifuged (5 min, 110 × g, 4 °C) and washed twice with PBS. The precipitate containing the cells was resuspended in 300 μl of ice-cold PBS, and added 700 μl of absolute ethanol at −20 °C, for fixing for one minute. Then the cells were washed twice with cold PBS. Cells were then incubated with 500 μl of warm PBS with RNase (Roche) for 30 min at 37 °C. Finally, 25 μl of propidium iodide (PI) (Sigma, 0.1 % (w/v)) in PBS were added. Cellular DNA content by PI incorporation was assessed (fluorescent emission between 562 and 588 nm) and can differentiate apoptotic cells (<2n), cells in the G0/G1, and cell proliferation (phase S / G2-M, > 2n). For this a FACScalibur cytometer and Cell Quest Pro software (Becton Dickinson) was used.

### Immunofluorescence

Cells were seeded at low density on circular crystals of 1 cm in diameter. Once treatment is completed, the cells were washed once with PBS and fixed with paraformaldehyde solution (4 % w/v), for 10 min at room temperature. Then washed with PBS, and incubated 5 min at room temperature with Triton X-100 (0.2 % v/v) in PBS in order to permeabilize the cells and permit labeling of intracellular antigens. To cushion the cellular autofluorescence, the cells were washed and incubated with a solution of NH_4_Cl (50 mM) for 15 min. at room temperature. After further washing, the nonspecific binding sites are blocked by incubation in a blocking solution of BSA (1 % w/v), 0.2 % Triton X-100 (v/v) in PBS supplemented with Tween-20 (0.05 % w/v) (PBST) for 45 min at room temperature. Incubation with primary antibodies was performed for 1.5 h at room temperature in a humid chamber. After washing with PBST, we incubated with secondary antibody for 45 min. After a final washing with PBST, the preparations were mounted on a slide with SlowFade mounted liquid Gold (Life Technologies) containing DAPI to visualize nuclei. Samples were generally observed to stabilize the following mounting liquid day, although stable preparations are stored at 4 °C in the dark for several weeks.

### RNA Extraction and Real-Time Quantitative Polymerase Chain Reaction

Total RNA was extracted from ECs by TRIzol method (Invitrogen, Barcelona, Spain) and quantified by absorbance at 260 nm in duplicate. Twenty nanogram of RNA were necessary to perform the reverse transcription reaction, for 15 min at 25 °C and 2 h at 37 °C, with the High Capacity cDNA Archive kit (Applied Biosystems, Foster City, CA). We evaluated the mRNA expression of genes involved in vascular dysfunction (eNOS and ICAM-1) and inflammation (MCP-1) on ECs. Amplification of GAPDH was used in the same reaction of all samples as an internal control. Gene-specific mRNA was subsequently normalized to GAPDH RNA. Quantitative reverse transcription-PCR was performed in 7500 Real-Time PCR device, and the relative quantification was performed with the Prism 7000 system SDS software (Applied Biosystems). The expression of these genes was analyzed by real-time quantitative PCR as described [17]. Thus, the amount of target, normalized to endogenous gene and relative to the control is given by 2^-ΔΔCt^ [ΔCt = Ct (target gene) – Ct (endogenous gene); ΔΔCt = ΔCt for any sample - ΔCt for the control].

### Statistical analysis

All values are expressed as mean +/− SEM. Data were analyzed using one-way analysis of variance, followed by a Bonferroni test if differences were noted (SPSS 15.0 program). The null hypothesis was rejected when the p value was less than 0.05.

## Results

### Characterization of cardiovascular cell lines

**Fig. 1:**
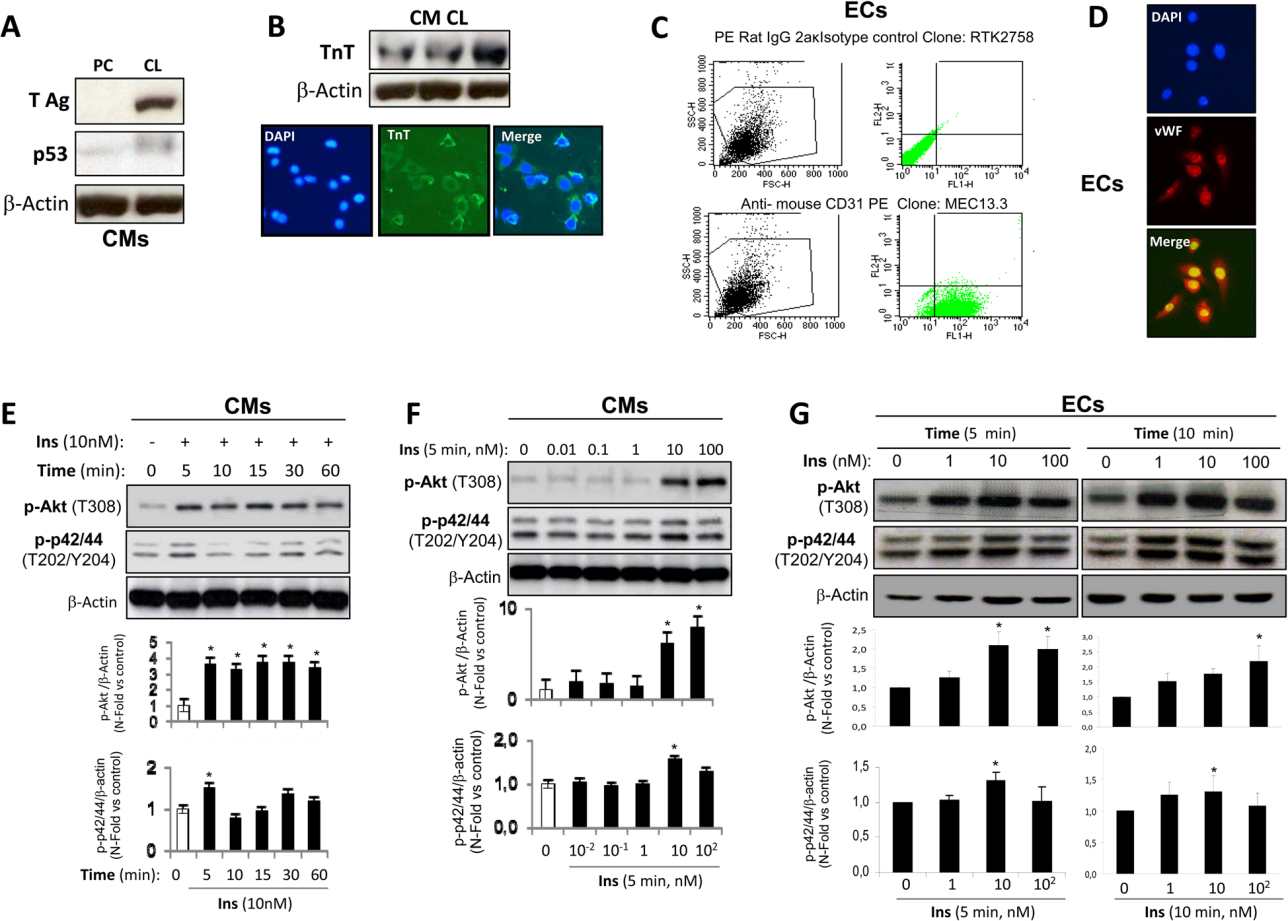
Characterization of cardiovascular lines. **a** Western blot analysis against AgT and p53 to check the immortalization performed in neonatal cardiomyocyte lines. β-actin was used as charge control. **b** Characterization of neonatal cardiomyocytes with a specific marker as TnT by Western blot or by immunofluorescence. Characterization of ECs with specific markers as PECAM-1 or CD31 by flow cytometry (**c**) or vWF by immunofluorescence (**d**). Western blot analysis of insulin signalling in CMs (**e**, **f**) and in ECs (**g**). *p < 0.05 vs. control

Firstly, we assured the CMs immortalization by Western blot analysis of T-antigen and p53 (Fig. 1a). Subsequently, we demonstrated the presence of the cardiomyocytes specific protein, troponin T (TnT), by Western blot and immunofluorescence (Fig. 1b). Moreover, we also characterized the ECs with CD31 antibody or PECAM-1 (Fig. 1c). By immunofluorescence, we detected the ECs specific protein, von Willebrand factor (vWF) (Fig. 1d).

Secondly, we analyzed insulin signaling in both cardiovascular cell lines. We observed that 10 nmol/L insulin induced an increase in Akt (T308) phosphorylation at 5 min. On the other hand, a significant increase of p42/44 (T202/Y204) phosphorylation was activated by 10 nmol/L insulin (Fig. 1e). Subsequently, an insulin dose–response curve showed that at 5 min the maximal activation of AKT was found at 100 nM insulin (Fig. 1f). Moreover, with the lowest insulin dose (1 nmol/L), the phosphorylation was induced in AKT and p42/44 at 5 and 10 min in ECs. A significant increase in AKT phosphorylation was observed at 10 and 100 nmol/L (Fig. 1g). VSMC lines used in this work were characterized and its insulin signaling shown in a previous work [18].

### Differential effect of oleic or palmitic acid on the cardiovascular cell insulin sensitivity

**Fig. 2:**
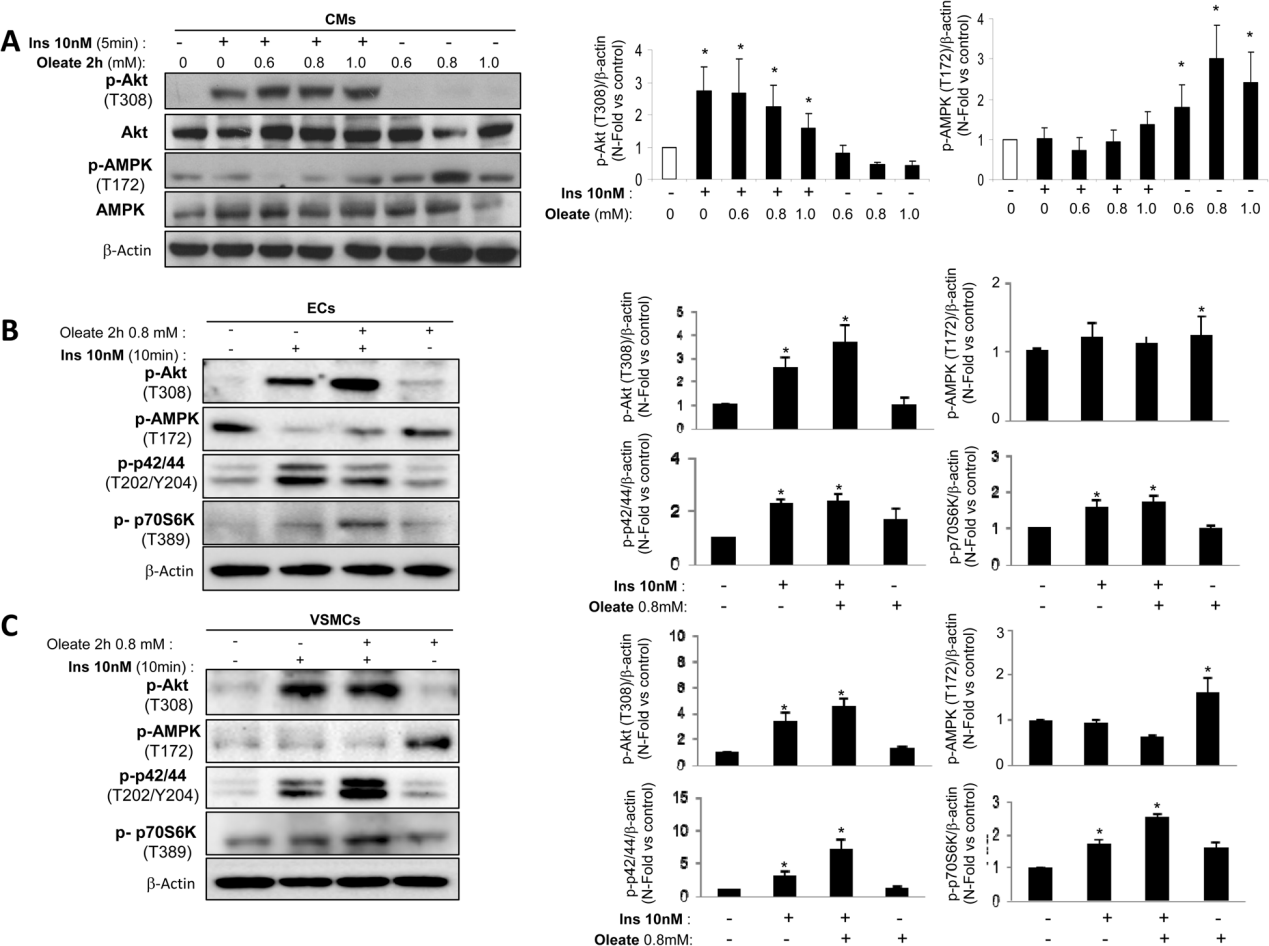
Oleate did not induce cardiovascular insulin resistance. Western blot analysis of phosphorylation de Akt (T308), AMPK (T172), p42/44 (T202/Y204) and p70S6K (T389) induced by insulin (10nM, 10 min) in presence or absence of oleate (2 h) in CMs (**a**), ECs (**b**) and VSMCs (**c**). β-actin was used as charge control. *p < 0.05 vs. control

**Fig. 3:**
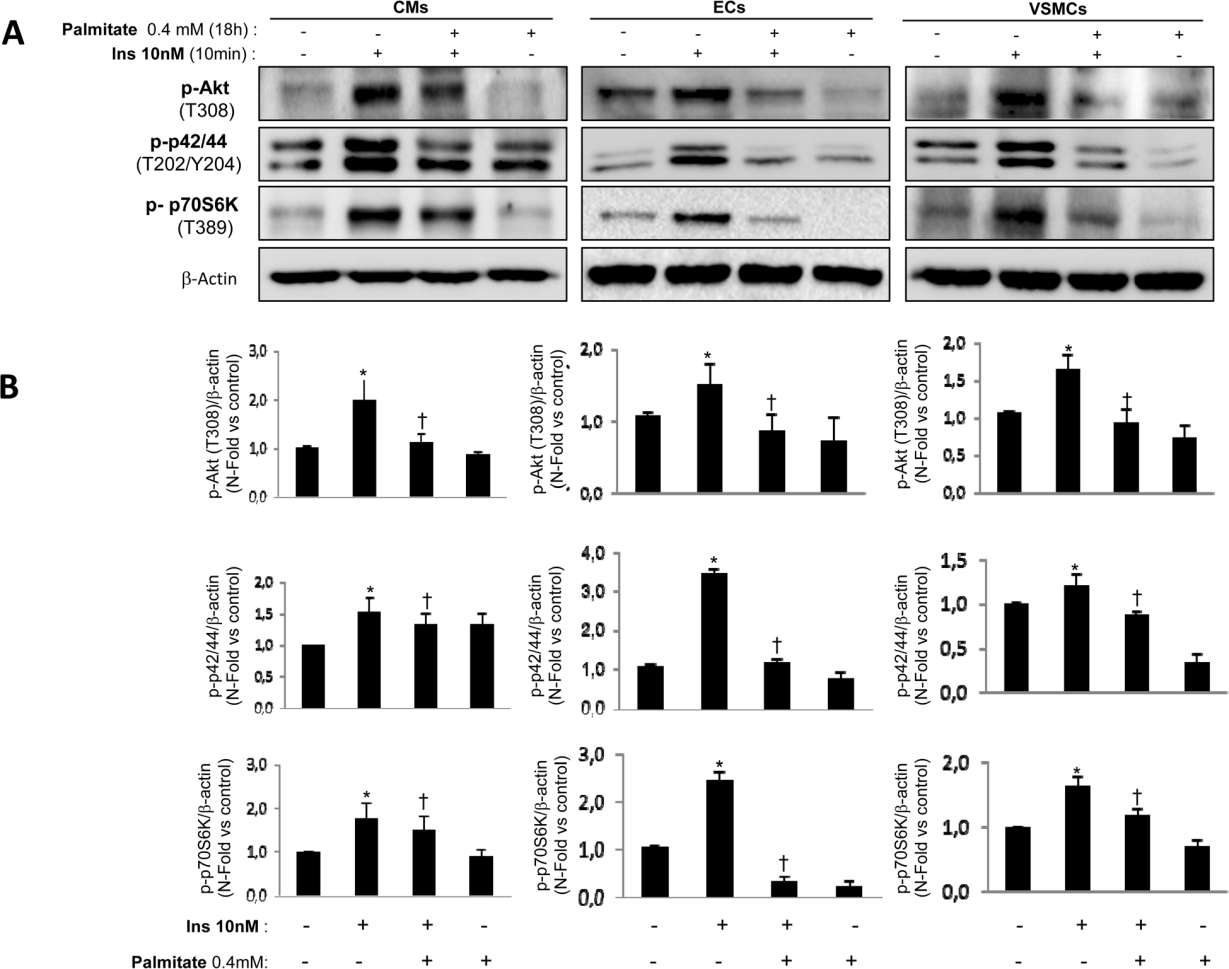
Palmitate induced cardiovascular insulin resistance. Western blot analysis (**a**) and its quantifications (**b**) of Akt phosphorylation (T308), p42/44 (T202/Y204) and p70S6K (T389) induced by insulin (10nM, 10 min) in presence or absence of oleate (2 h) in CMs, ECs and VSMCs. β-actin was used as charge control. *p < 0.05 vs. control; †*p* < 0.05 vs. stimulus

Firstly, we studied the oleate effect on insulin sensitivity. Thus, cardiomyocytes were treated with oleate at 0.6, 0.8 or 1 mmol/L for 2 h before insulin stimulation. We observed that insulin stimulation significantly increased Akt phosphorylation for each dose of oleate used. Under the same conditions, Akt activation was accompanied by the inactivation of AMPK phosphorylation (Fig. 2a). However, we observed AMPK activation in absence of insulin (Fig. 2a). In vascular cells, 0.8 mmol/L oleate did not prevent Akt, p42/44 and p70S6K phosphorylation or AMPK dephosphorylation in response to insulin (Fig. 2b, c). In addition, oleate induced AMPK activation in the absence of insulin in vascular cells (Fig. 2b, c). Overall, oleate treatment for 2 h did not produce insulin resistance in the cardiovascular cells studied. These results were confirmed by the dose–response effect of oleate at 18 h on Akt phosphorylation in the presence of insulin (Additional file 1: Figure S1). At this stage, we confronted these data regarding oleate with palmitate effect on insulin sensitivity. For that purpose, we chose 0.4 mmol/L palmitate dose as previously described in myocytes [15]. Palmitate (0.4 mmol/L) treatment did not prevent Akt or p42/44 phosphorylation induced by insulin for 2 or 6 h in CMs. However, prolonged palmitate treatment for 18 or 24 h induced insulin resistance (Additional file 2: Figure S2). At this stage, 0.4 mmol/L palmitate for 18 h significantly induced insulin resistance as revealed by Akt, p42/44 and p70S6K phosphorylation in response to insulin in all the vascular cells studied (Fig. 3).

**Fig. 4:**
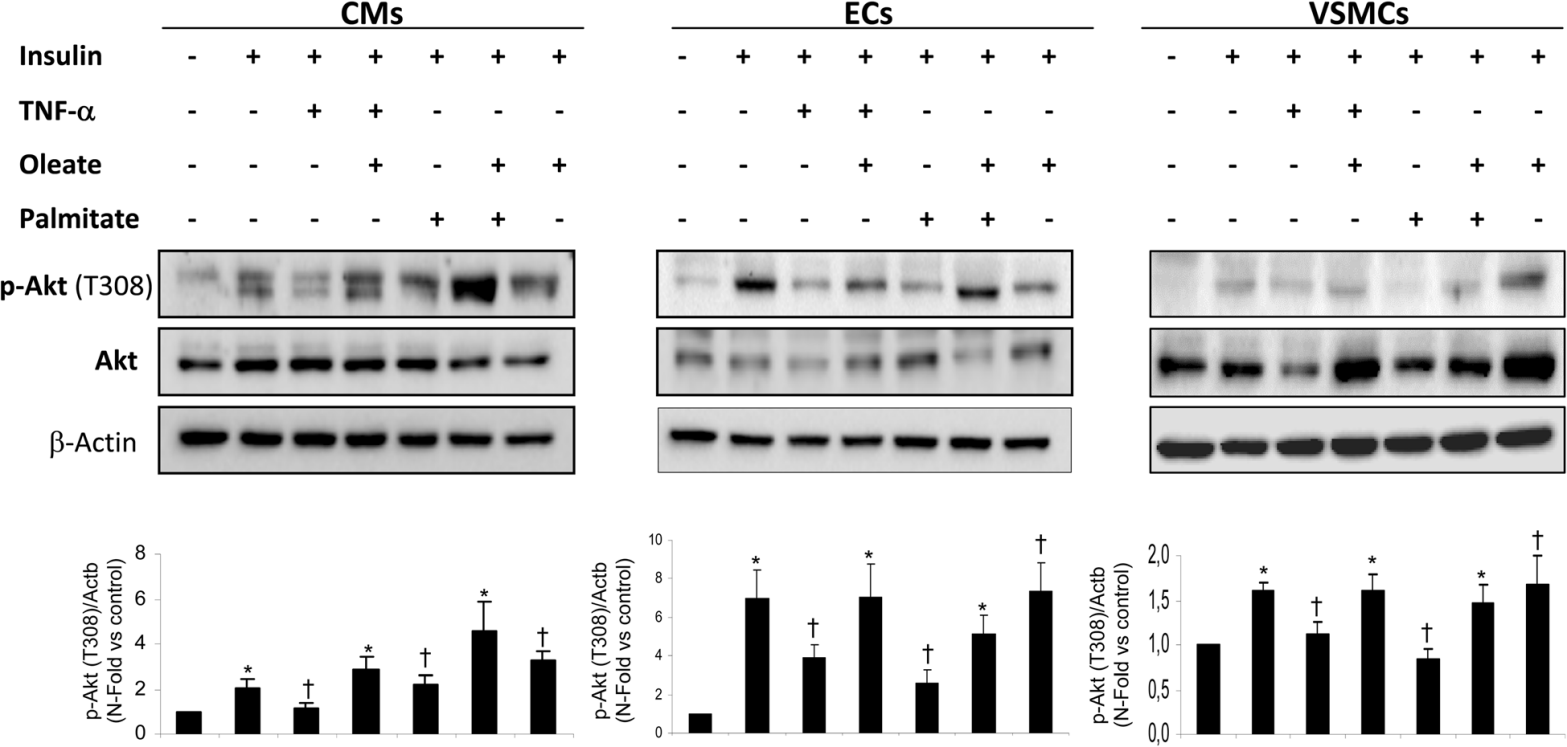
Oleate protected cardiovascular insulin resistance induced by TNF-α or palmitate. Western blot analysis of phosphorylation of Akt (T308) induced by insulin (10nM, 10 min) and /or palmitate (0.4 mM, 18 h) or TNF-α (10 ng/mL, 2 h) in presence or absence of oleate in CMs, ECs and VSMCs. β-actin was used as charge control. *p < 0.05 vs. control; †*p* < 0.05 vs. stimulus

**Fig. 5:**
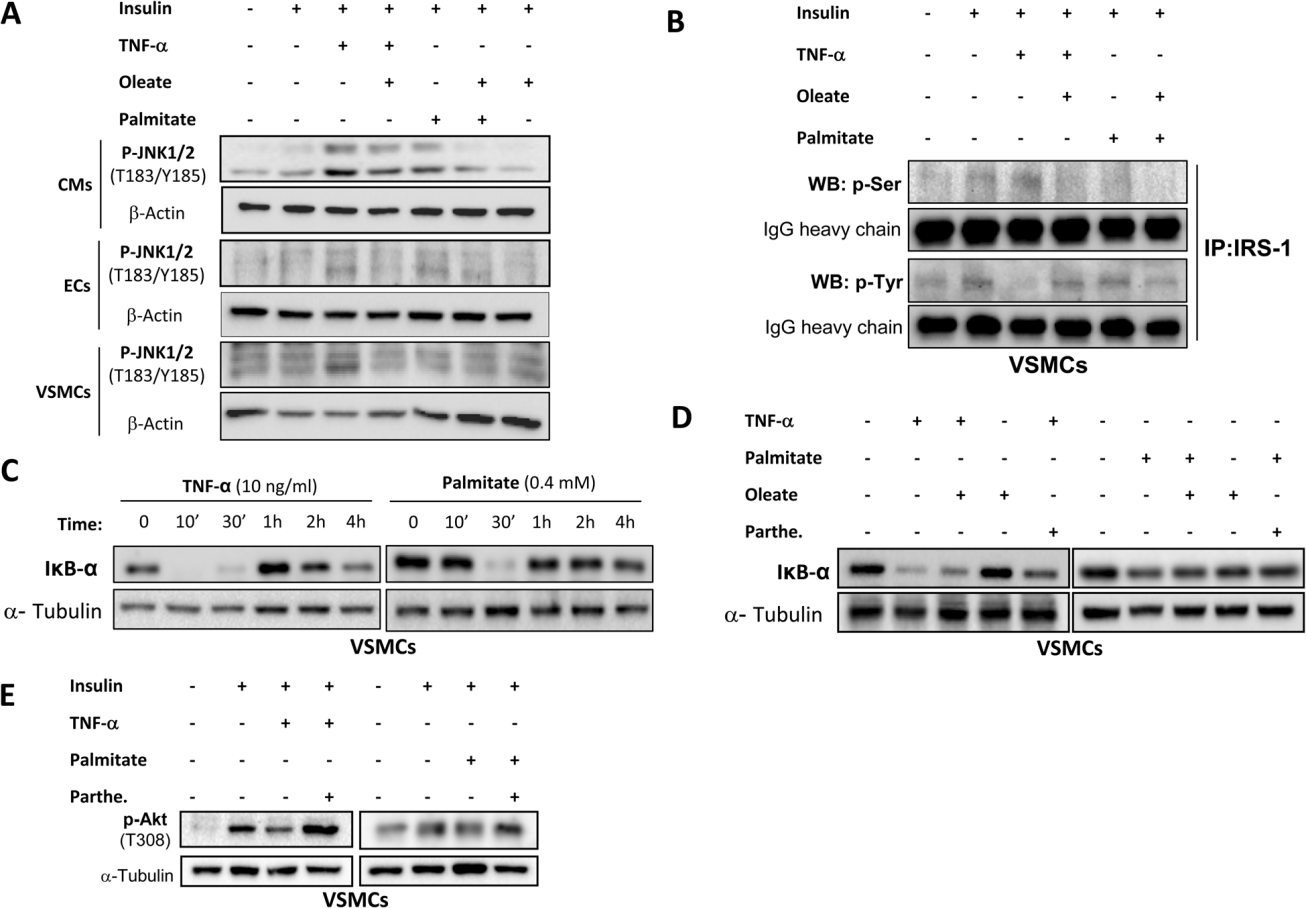
Modulation of JNK-1/2 and NF-κB pathway by oleate. **a** Western blot analysis of JNK-1/2 phosphorylation in CMs, ECs and VSMCs. **b** Effect of oleate in the Ser and Tyr phosphorylation of IRS-1 in VSMCs. Effect of TNF-α or palmitate in IκBα levels in absence (**c**) or presence (**d**) of oleate in VSMCs. **e** Effect of parthenolide in the phosphorylation of Akt in VSMCs. β-actin was used as charge control

We also demonstrate that TNF-α at 2 h induced vascular insulin resistance as palmitate did at 18 h (Fig. 4). The pretreatment with oleate prevented the inhibition by TNF-α or palmitate on Akt phosphorylation induced in response to insulin in cardiovascular cells (Fig. 4). Beside the previously proposed mechanism, according to which oleate may be protecting against cardiovascular damage induced by TNF-α by UCP-2 expression [19], we studied the NF-κB signaling pathway. Thus, we observed that TNF-α as well as palmitate increased JNK1/2 phosphorylation in all the vascular cells studied. However, the pretreatment with oleate reduced JNK phosphorylation induced by TNF-α or palmitate (Fig. 5a). When JNK1/2 was activated by TNF-α, there was an increase in IRS-1 serine phosphorylation and a decrease in IRS-1 tyrosine phosphorylation. Pretreatment with oleate prevented serine phosphorylation and maintained Tyr phosphorylation on IRS-1 induced by TNF-α or palmitate (Fig. 5b). Another mechanism that might be modulated by oleate, could be mediated by NF-κB. Thus, we observed that TNF-α, from 10 to 30 min and palmitate at 30 min were able to reduce the levels of IκB-α (Fig. 5c). Subsequently, we observed that oleate was able to partially prevent IκB-α degradation induced by TNF-α or palmitate. More importantly, parthenolide, an inhibitor of NF-κB [20], prevented IκB-α degradation induced by TNF-α (Fig. 5d) and precluded insulin resistance on Akt phosphorylation induced by TNF-α or palmitate in VSMCs (Fig. 5e).

### Protective role of oleate in the endothelial dysfunction markers and in the inflammatory response

We decided to study the effect of several cytokines involved in the inflammatory process, such as TNF-α, IL-6 and IL-1β on some markers of endothelial dysfunction and activation in ECs and the possible protective effect of the oleate against those stimuli.

**Fig. 6:**
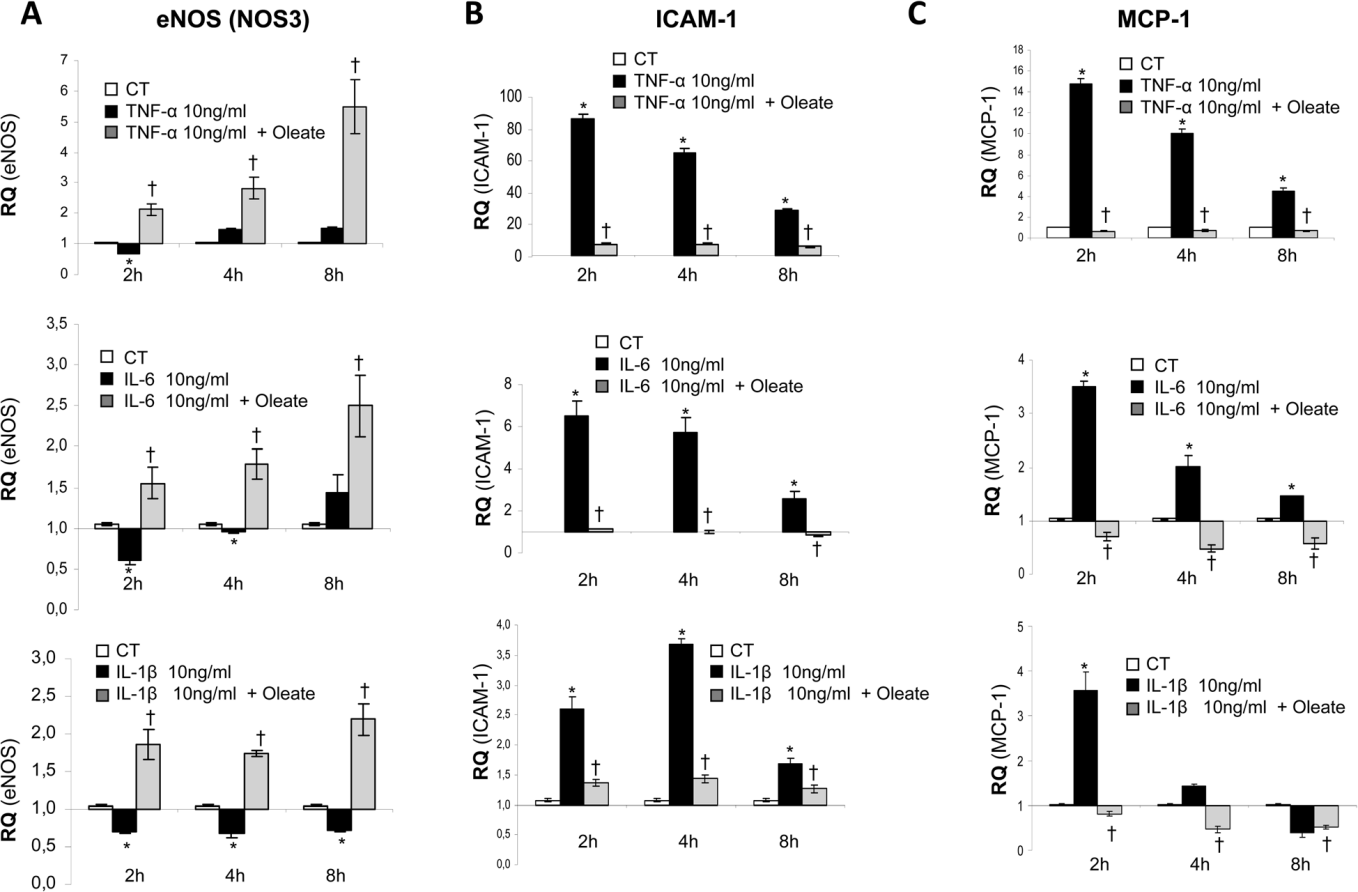
Effect of oleate in eNOS, ICAM-1 and MCP-1 mRNA expression in ECs. qRT-PCR analysis of eNOS (**a**), ICAM-1 (**b**) and MCP-1 (**c**) mRNA expression induced by TNF-α, IL-6 and IL-1β in presence or absence of oleate in ECs. *p < 0.05 vs. control; †*p* < 0.05 vs. stimulus

First, we observed that the treatment with oleate prevented eNOS mRNA reduction induced by TNF-α, IL-6 or IL-1β in ECs (Fig. 6a). We also observed that the pretreatment with oleate significantly reduced ICAM-1, and MCP-1 expression induced by the pro-inflammatory cytokines in ECs (Fig. 6b, c).

### Protective effect of the oleate on the VSMCs proliferation

**Fig. 7:**
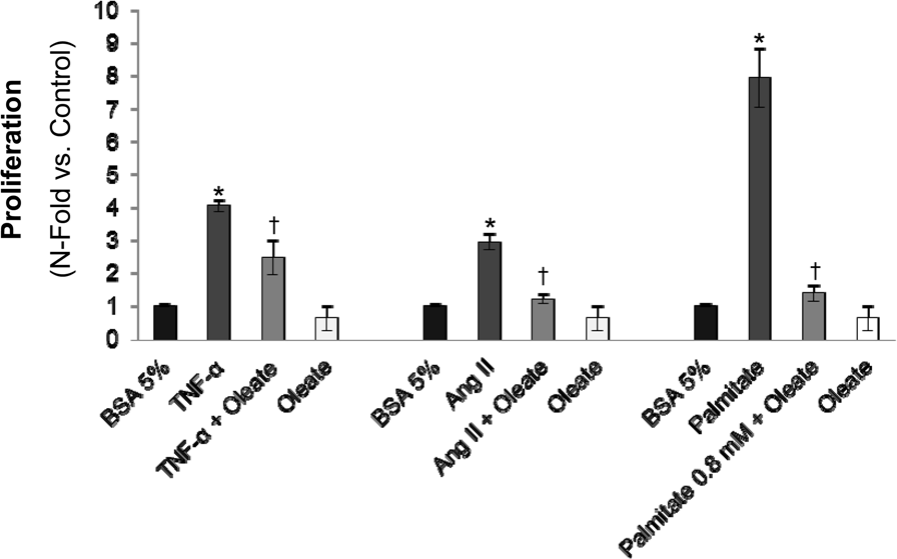
Effect of oleate in VSMCs proliferation. Rates of proliferation measured by BrdU incorporation in response to TNF-α, angiotensin II or palmitate in presence or absence of oleate. *p < 0.05 vs. control; †*p* < 0.05 vs. stimulus

One of the factors that contribute to the early growth of atherosclerotic plaque is the migration and proliferation of VSMCs. On this regard, pretreatment with oleate mostly prevented the rate of proliferation induced by TNF-α, Ang II or palmitate (Fig. 7).

### Protective effect of oleate on the VSMCs apoptosis

**Fig. 8:**
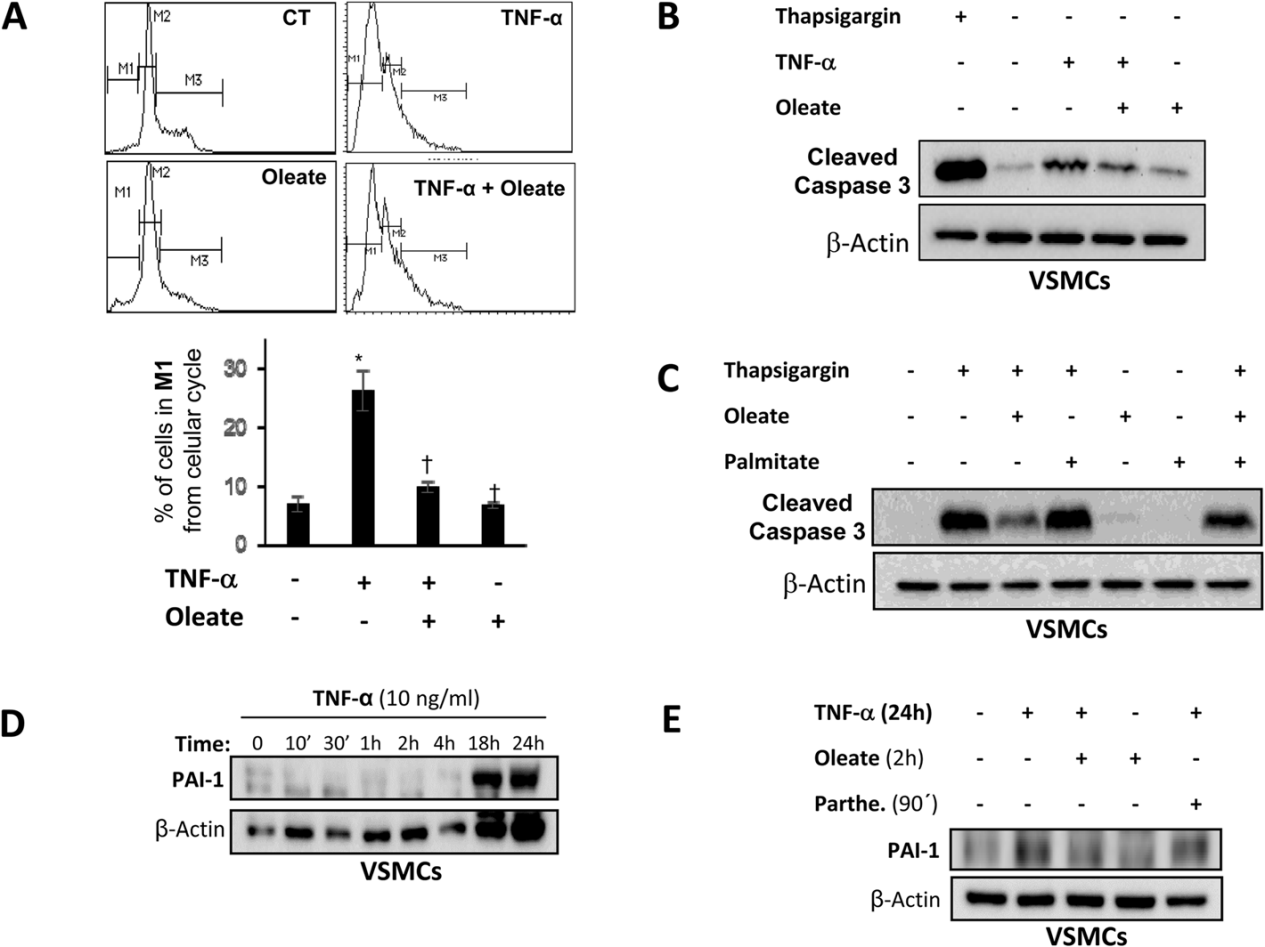
Effect of oleate in VSMCs apoptosis. Effect of oleate in VSMCs apoptosis induced by TNF-α measured by cellular cycle (**a**) or by Western blot analysis of active caspase 3 (**b**) or induced by thapsigargin by Western blot analysis (**c**). *p < 0.05 vs. control; †*p* < 0.05 vs. stimulus. **d** Western blot analysis of PAI-1 levels induced by TNF-α at different time points. **e** Effect of oleate and parthenolide in PAI-1 levels induced by TNF-α. β-actin was used as charge control

One of the described mechanisms and directly involved in the instability and rupture of atherosclerotic plaque, is the increase of fibrous cap VSMCs apoptosis, weakening it and triggering the acute event [21, 22]. For this purpose, we explored if oleate could improve the plaque stability, reducing VSMCs apoptosis. First, we observed that the treatment with TNF-α significantly increased the percentage of cells in G0/G1 phase in comparison to the control. In addition, oleate *per se* does not alter the cellular cycle in VSMCs. More importantly, the pre-treatment with oleate significantly reduced the percentage of apoptotic cells induced by TNF-α (Fig. 8a). On this regard, we analysed active caspase-3 protein levels induced by several stimuli. As a positive control of apoptosis, we used 1 μg/ml thapsigargin for 30 min that induced a high expression of active caspase-3. Thus, TNF-α increased the expression of active caspase-3 as compared with the control. In the presence of oleate, the expression of caspase-3 induced by TNF-α was significantly impaired (Fig. 8b). In addition, oleate also impaired active caspase-3 levels induced by thapsigargin (Fig. 8c). However, palmitate did not show any protective effect on the expression of active caspase-3 induced by thapsigargin. Conversely, oleate impaired the rate of apoptosis induced by thapsigargin in the presence of palmitate (Fig. 8c).

### Protective effect of oleate on thrombogenesis in vascular cells

Elevated levels of PAI-1 are associated with increased cardiovascular and thrombotic events [23]. On this regard, TNF-α induces the expression of PAI-1 between 18 and 24 h in VSMCs (Fig. 8d). At this stage, we explored whether or not TNF-α could be increasing the production of PAI-1 in VSMCs through the activation of NF-κB. For this purpose, we observed that when VSMCs were pretreated with parthenolide and therefore NF-κB pathway was inhibited, the production of PAI-1 induced by TNF-α was partially reduced (Fig. 8e). Next, we decided to explore the effect of oleate on the expression of PAI-1 induced by TNF-α in VSMCs. First, we observed that oleate *per se* did not increase PAI-1 protein levels. However, treatment with oleate mostly prevented the elevation of PAI-1 induced by TNF-α (Fig. 8e).

## Discussion

Over the past decades, the prevalence of obesity and metabolic syndrome (MetS), and its underlying risk of developing cardiovascular disease (CVD), has increased worldwide becoming a public health problem [24]. It is known that subjects with MetS have an increased risk of developing type 2 diabetes, in turn, these people tend to be overweight or obese, with high levels of circulating FFA. This increase contributes to the complications of obesity, such as insulin resistance, excessive fat accumulation in peripheral tissues [25] and alterations in cardiac function [26, 27], among others. Additionally, increased levels of circulating FFAs are recognized as a predictor of myocardial infarction [28].

FFAs may act as either pro- or anti-inflammatory agents depending on the chemical structure [29]. SFAs, such as palmitic acid, have been associated with adverse cardiovascular effects [30, 31]. However, PUFAs, such omega-3 FAs, improve triglyceride levels and reduce the risk of CVD by their anti-inflammatory properties [28]. Moreover, it has recently been reported that there is not association between dietary omega-3 FA and the risk of MetS [32]. Similarly to PUFAs, MUFAs, such as oleic acid, has been associated with antiproliferative effects on cancer and decreased risk of CVD [13, 33]. Translational studies as PREDIMED have recommended from Mediterranean diet, the use of virgin olive oil where its main fatty acid is oleate due to decreased CVD and improve irrigation in the treatment of Type 2 Diabetes Mellitus [10, 33]. However, the molecular mechanisms by which oleate exerts its protective role in vascular cells is not fully understood. Our results show that oleate has a differential beneficial cardiovascular effect with respect to other SFAs as palmitate. So far the evidence pointed to the long-chain SFA palmitate induced insulin resistance in tissues such as adipocytes and skeletal muscle [34, 35]. We showed that in cardiovascular cells, palmitate also induces insulin resistance. In this regard, others authors also demonstrated that SFAs stimulated the fatty-acid uptake in detriment of glucose assimilation in cardiomyocytes [36] and *in vivo*, SFAs intake is associated with most cardiac remodeling [37]. However, we have shown that oleate did not generate insulin resistance in any studied cardiovascular cell lines. Similarly to our results in cardiovascular cells, oleate was also able to prevent insulin resistance in the myotubes through activation of PI3K [35] or by a mechanism dependent of AMPK [15]. In addition, in the pancreatic β-cells, it has been described that oleate induced activation PI3K and PKB pathway and prevented apoptosis [38]. On the other hand, other authors have described that both oleate and palmitate induced insulin resistance in primary hepatocytes, associated with the accumulation of dyacilglycerols and/or ceramide [39] or a diet rich in UFAs increased total adiposity without impairing cardiovascular parameters [40].

In the present work, we have shown that oleate did not induce insulin resistance in the context of cardiovascular cells and moreover it is capable of protecting against insulin resistance induced by palmitate or TNF-α. Just recently, we proposed that oleate induced an increase of UCP-2 levels and this might be one of the mechanisms by which oleate protects against the deleterious action of palmitate on vascular cells [19]. Additionally, we have now hypothesized that palmitate or TNF-α could be activating different pathways such as JNK and NF-κB in cardiovascular cells. One of the mechanisms that may be involved in the deleterious effects of SFAs is that they can act as ligands for TLR, and activate different signaling pathways involved in the inflammatory response [41, 42]. Thus, it has been described that palmitate through TLR2 may induce insulin resistance in myotubes, inducing activation of NF-κB, JNK and p38 [2] and the impairment of vasodilator actions of insulin [43]. In our work, both palmitate and TNF-α activated JNK pathway in all three cardiovascular cell lines. However, pretreatment with oleate substantially decreased activation of JNK1/2. These results confirm that oleate prevents insulin resistance avoiding IRS-1 Ser phosphorylation and maintaining IRS-1 Tyr phosphorylation in presence of TNF-α or palmitate, favoring the activation of the PI3K pathway.

Our results also suggest that other mechanism by which oleate prevents insulin resistance in VSMCs is the modulation of NF-κB activation. We demonstrate that TNF-α or palmitate activate NF-κB, decreasing the levels of IκB-α [44]. However, the reduction of NF-κB by oleate might be implicated in the protective role of oleate against insulin resistance induced by the TNF-α or palmitate. We also checked that parthenolide, an inhibitor of NF-κB [20], prevented IκB-α degradation and therefore the activation and translocation of NF-κB to the nucleus in addition to prevent insulin resistance induced by TNF-α or palmitate.

In the literature, it has also been described the differential correlation among oleate and palmitate with inflammasomes. Thus, the proinflammatory effect of SFAs through a caspase-1/ASC/NLRP3-dependent pathway [45] might justify that SFA-rich diets increase IL-1β production and other inflammatory processes related to IL-1β [45, 46]. However, anti-inflammatory actions of MUFAs prevent activation of NLRP3 inflammasome induced by SFAs in human monocytes/macrophages [47], supporting the beneficial effects of MUFAs in Mediterranean diet [10]. Moreover, high MUFAs diet or replacement of SFAs for MUFAs induce changes in abdominal fat distribution, improve insulin sensitivity [48, 49], and postprandial oxidative stress in patients with metabolic syndrome [50].

It is known that during processes such as obesity or atherosclerosis, there are increased levels of proinflammatory cytokines such as IL-6 and TNF-α, which are capable to activate NF-κB in different tissues. At the same time, this proinflammatory cytokines activate other pro-inflammatory and chemotactic agents creating a cycle of self-maintaining inflammation [51]. The permanence of the inflammatory condition in the vasculature can induce endothelial dysfunction and activation with an increase in the expression of chemotactic factors and adhesion molecules such as ICAM-1 and VCAM-1 [52]. Thus, our work suggests that oleate has also a protective role in the activation and endothelial function in addition to the inflammatory response. These findings correlate with the positive effect of oleate found in human VSMCs and ECs [53, 54].

TNF-α is capable of activating two opposite mechanisms, cell survival and cell death simultaneously [55, 56]. Thus, we found that TNF-α, but not palmitate, significantly induced an increase in the percentage of cells found in the G0/G1 phase of the cell cycle. However, treatment with palmitate has a different behavior between the two vascular lines: decreases the viability of ECs, but favors the proliferation of VSMCs, and may play an important role in the initiation and progression of atherosclerosis. On this regard, saturated, but not unsaturated, fatty acids induce apoptosis of human coronary artery endothelial cells via nuclear factor-kappaB activation [57]. On the other hand, we demonstrated that oleate exerted a protective role against the proliferation of VSMCs stimulated by TNF-α, Ang II, or palmitate, contributing to the prevention of the growth of atherosclerotic plaque. Converserly, palmitate induced changes in VSMCs phenotype promoting formation of atherosclerotic plaque [31].

Increased apoptosis of VSMCs is one of the mechanisms directly involved in the instability and rupture of atherosclerotic plaques, and underlying complications such as thrombosis [22, 23]. We propose that pretreatment with oleate has a protective role against apoptosis in VSMCs owing to oleate pretreatment significantly reduced the percentage of apoptotic cells and active caspase-3 protein levels induced by TNF-α. It has been previously described that oleate modulates proapoptotic proteins expression as Bax, caspase-3 and PARP cleavage in cardiomyocytes [58]. Thus, we confirm that oleate exerts a protective role against apoptosis in VSMCs, decreasing the activation of caspase-3, induced by TNF-α or thapsigargin that induces stress in the endoplasmic reticulum [59].

Elevated levels of PAI-1 are associated with increased cardiovascular and thrombotic events [23]. We found that oleate reduces PAI-1 protein levels induced by TNF-α in VSMCs, and we show that increased PAI-1 induced by TNF-α is through activation of NF-κB, given that parthenolide decreases PAI-1 levels induced by TNF-α in VSMCs. We suggest that oleate exerts its protective effects through inhibition of NF-κB pathway, and also improves the thrombogenesis promoting the fibrinolysis by inhibition of PAI-1.

## Conclusions

In conclusion, our results show that oleic acid has a beneficial effect at cardiovascular level as compared with saturated fatty acids such as palmitic acid. Thus, oleate protects against cardiovascular insulin resistance, improves endothelial dysfunction, inflammation and finally, reduces proliferation and apoptosis in VSMCs that may contribute to an ameliorated atherosclerotic process and its stability. These processes may be mediated by inhibition of JNK-1/2 and NF-κB pathways.

## Additional files

Additional file 1: Figure S1. Oleate did not induce vascular insulin resistance for a long time. Western blot analysis of Akt (T308) phosphorylation in ECs and VSMCs stimulated with insulin (10nM, 10 min) with or without oleate (0.6, 0.8 or 1 mM for 18 h). β-actin was used as charge control. Additional file 2: Figure S2. Effect of palmitate for different times in insulin signaling in neonatal cardiomyocytes. Representative gels (A) and its quantifications (B and C) of Western blot analysis of Akt (T308) and p42/44 (T202/Y204) phosphorylation in cardiomyocytes (CMs) stimulated with insulin (10nM, 10 min) with or without palmitate (0.4 mM for 2, 6,18 or 24 h). β-actin was used as charge control. **p* < 0.05 vs. control; †*p* < 0.05 vs. stimulus.

## Abbreviations

AKT: Protein kinase A
CMs: Cardiomyocyte cells
ECs: Endothelial cells
eNOS: Endothelial nitric oxide synthase
ERK-1/-2: Extracellular signal-regulated kinase
ICAM-1: Intercellular adhesion molecule 1
iNOS: Inducible nitric oxide synthase
IRS-1: Insulin receptor substrate 1
MCP-1: Monocyte chemoattractant protein-1
NF-κB: Nuclear factor kappa B
OA: Oleic acid
PA: Palmitic acid
PAI-1: Plasminogen activator inhibitor-1
qRT-PCR: Quantitative real time reverse transcription polymerase chain reaction
VSMCs: Vascular smooth muscle cells
