# Supplementary figures and images for: Protective role of oleic acid against cardiovascular insulin resistance and in the early and late cellular atherosclerotic process
Source: Cardiovasc Diabetol. 2015 Jun 10;14:75. doi: 10.1186/s12933-015-0237-9 (PMC4475625; doi:10.1186/s12933-015-0237-9)

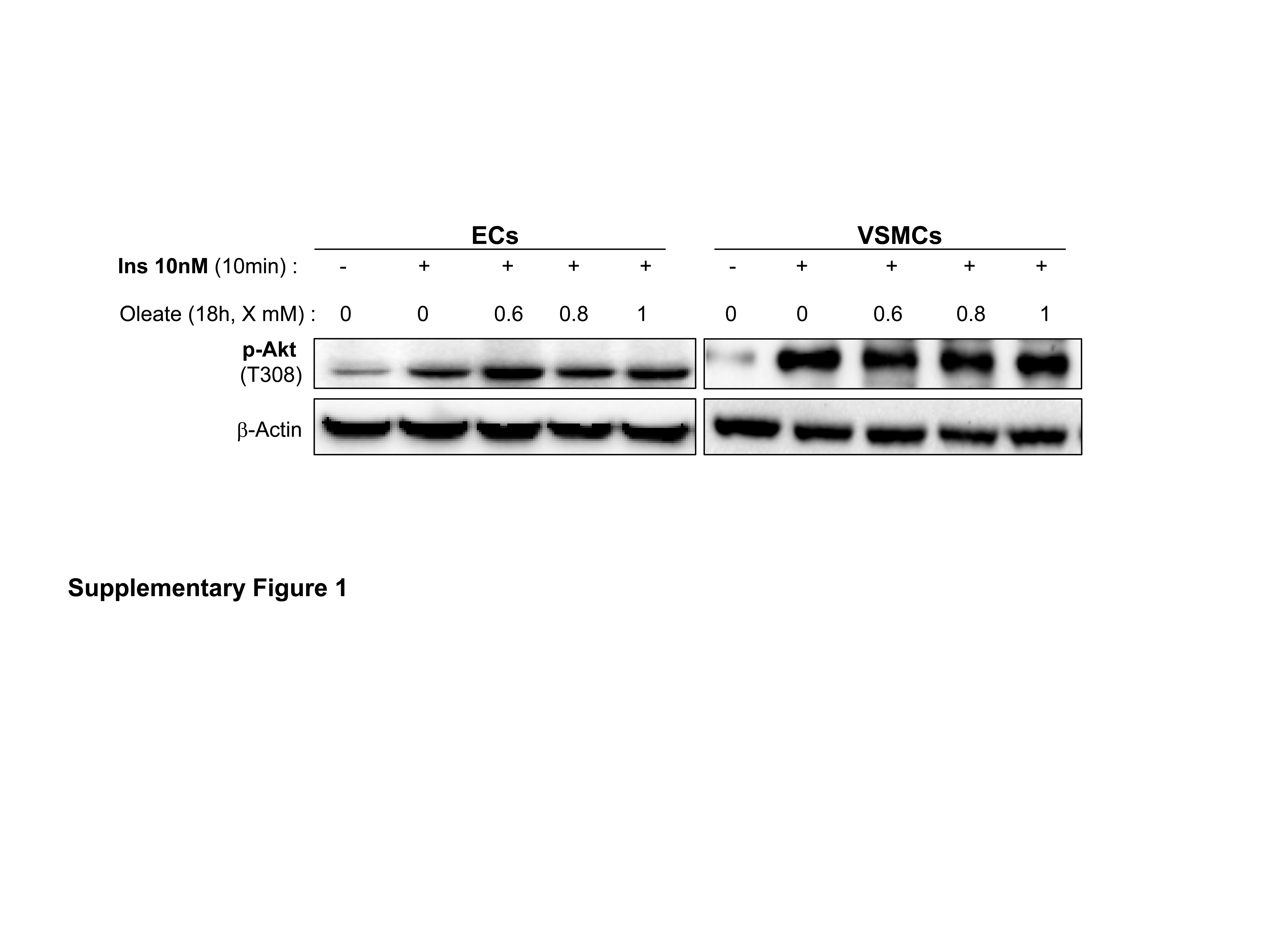

Supplement: Additional file 1: Figure S1. — Oleate did not induce vascular insulin resistance for a long time. Western blot analysis of Akt (T308) phosphorylation in ECs and VSMCs stimulated with insulin (10nM, 10 min) with or without oleate (0.6, 0.8 or 1 mM for 18 h). β-actin was used as charge control. [file 12933_2015_237_MOESM1_ESM.tif]

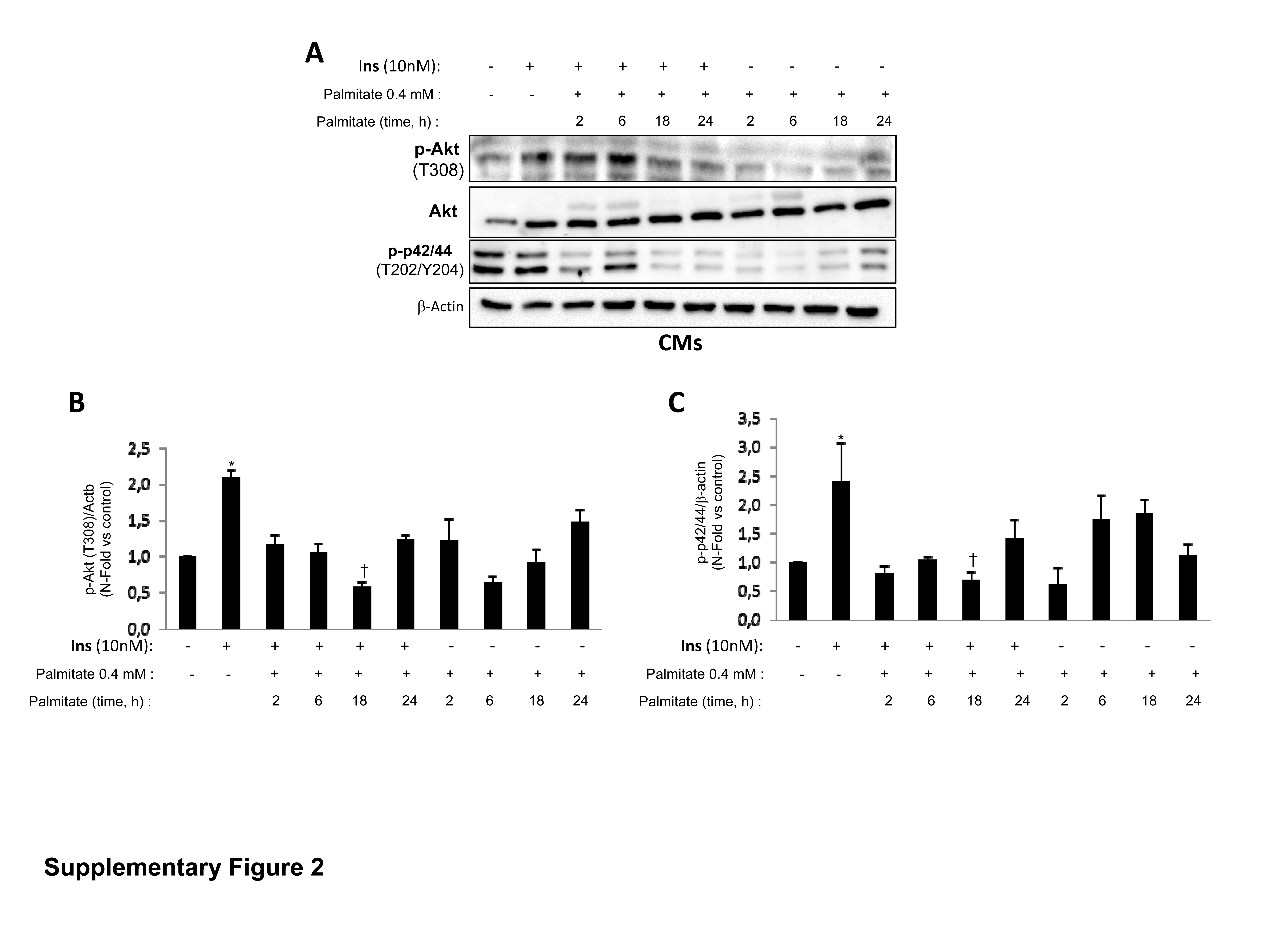

Supplement: Additional file 2: Figure S2. — Effect of palmitate for different times in insulin signaling in neonatal cardiomyocytes. Representative gels (A) and its quantifications (B and C) of Western blot analysis of Akt (T308) and p42/44 (T202/Y204) phosphorylation in cardiomyocytes (CMs) stimulated with insulin (10nM, 10 min) with or without palmitate (0.4 mM for 2, 6,18 or 24 h). β-actin was used as charge control. *p < 0.05 vs. control; †p < 0.05 vs. stimulus. [file 12933_2015_237_MOESM2_ESM.tif]
